# Maine Medical School

**Published:** 1860-01

**Authors:** 


MAINE MEDICAL SCHOOL.—Dr. Chas. A. Lee having resigned the chair of Materia Medica in this institution, Dr. Israel T. Dana has been elected in his place.

